# The relationship between premenstrual syndrome and personality traits in university students

**DOI:** 10.1590/1806-9282.20231679

**Published:** 2024-08-16

**Authors:** Figen Alp Yılmaz

**Affiliations:** 1 Alanya Alaaddin Keykubat University, Faculty of Health Sciences, Department of Nursing - Antalya, Turkey.

**Keywords:** Personality traits, Premenstrual syndrome, Students

## Abstract

**OBJECTIVE:**

This study was conducted to determine the relationship between personality traits and premenstrual syndrome in university students

**METHOD:**

This cross-sectional study was conducted with 616 female university students between February and June 2020.

**RESULTS:**

The Premenstrual Syndrome Scale score was determined as a mean of 125.40±25.41. According to linear regression analysis, extrovert/introvert personality traits, emotional balance/neuroticism, and consistent/inconsistent personality traits were statistically significant predictive factors of premenstrual syndrome.

**CONCLUSION:**

The results of this study demonstrated that two-thirds of the students had premenstrual syndrome and personality traits affected premenstrual syndrome. It is recommended that attention should be paid to personality traits when coping with premenstrual syndrome.

## INTRODUCTION

Premenstrual syndrome (PMS) is the umbrella term for a group of physical, emotional, and psychological symptoms of varying severity that occur in the late luteal phase of the menstrual cycle and end after the onset of menstruation^1^. Although the specific symptoms experienced vary greatly between individuals, commonly reported physical symptoms of PMS include breast tenderness, bloating, fatigue, headaches, and appetite changes. Emotional and psychological symptoms may include mood swings, irritability, anxiety, depression, and difficulty concentrating^2–4^. PMS symptoms can cause changes in body image and life activities, loss of workforce, economic losses, increased likelihood of accidents, decreased self-confidence, alcohol/drug use, increased tendency for crimes, deterioration in social relationships, and decreased academic performance^5,6^.

Studies conducted in Turkey and other countries have shown that PMS is a common problem among women^3,7,8^. PMS is considered an important health problem due to its prevalence and the negative effects of the symptoms on daily living activities, academic/professional life, and the individual's productivity^9^. Although it is many years since PMS was first described, the mechanism behind its occurrence is not yet fully understood^4^. The fact that most PMS symptoms are psychological and behavioral suggests that they may be related to personality structure^10^. Individuals respond to events in line with their personality traits and determine ways to cope accordingly. There are studies in the literature showing that PMS is related to the personality structure of women, and it has been determined that the personality traits of "Neuroticism" and "Agreeableness" affect PMS and that neurotic personality is a predictor of PMS^11^. Determining the relationship between personality structures and PMS may be beneficial in coping with and treating premenstrual complaints. Therefore, the aims of this study were as follows: (1) to determine the frequency of PMS in university students, (2) to determine the level of PMS and personality traits in university students, (3) to examine the relationship between PMS and personality traits, and (4) to identify significant factors predicting PMS in university students.

## METHODS

This descriptive, cross-sectional study was conducted between February and June 2020 at a university in the Central Anatolia region of Yozgat. The study sample consisted of 616 female students. The sample size was calculated with power analysis to provide an effect size of 0.05 and a 90% confidence interval determined with a 5% error level. The study group was formed of female students aged ≥18 years, who had a regular menstrual cycle and no known physical or psychological disorders. Students with any psychiatric illness were excluded from the study.

### Data collection

Data were collected with the Personal Information Form, Premenstrual Syndrome Scale (PMSS), and Cervantes Personality Scale (CPS).

Personal Information Form: This form contained questions to determine the socio-demographic characteristics of the university students.

### Premenstrual Syndrome Scale

This 44-item scale with Likert-type responses was developed by Gençdoğan in 2006. Depressive affect, anxiety, fatigue, irritability, depressive thoughts, pain, appetite change, sleep change, and bloating are the sub-dimensions of the scale. The total score that can be obtained from the scale ranges from 44 to 220 points. A total PMSS score of ≥111 indicates the presence of PMS. In the original study, the Cronbach's alpha coefficient of the PMSS items was 0.75, while that of the subscales was between 0.75 and 0.91. In this study, the Cronbach's alpha coefficient was 0.88 for PMSS items and varied between 0.57 and 0.65 for subscales^12^.

### Cervantes Personality Scale

This scale was developed by Castelo-Branco et al. to evaluate the personality traits of women, and validity and reliability studies were conducted by Bal and Şahin. Each question in the scale is answered according to the individual's own experiences. The scale is a six-point Likert-type scale consisting of 20 items with three subscales (Extroversion/introversion, Emotional balance/neuroticism, Consistency, and inconsistency). As the mean scores from the sub-dimensions decrease, the personality traits of being extroverted, emotional balance, and consistency are seen more frequently. The Cronbach's alpha reliability coefficient of the CPS has been calculated as α=0.97 for the extroversion/introversion dimension, α=0.81 for the emotional balance/neuroticism dimension, and α=0.71 for the consistent/inconsistent dimension^12^. In this study, the reliability coefficient was found to be 0.82 for extroversion/introversion, 0.78 for emotional balance/neuroticism, and 0.69 for consistency/inconsistency.

### Ethical considerations

This study was approved by Yozgat University Social Sciences Ethics Committee. Before starting the data collection process, written permission was obtained from the university where the research was conducted. Written informed consent for voluntary participation in the research was provided by all students.

### Statistical analysis

After examining the normality distribution of the data, descriptive statistical methods such as frequency, percentage, mean, and standard deviation were used in the analyses. Differences between independent groups were analyzed using the independent-samples t-test and one-way ANOVA test. Pearson correlation analysis was used to evaluate the relationships between the scale average scores. Multiple linear regression analysis was performed to evaluate the relationship between the PMSS scores and the subscales of the CPS. The reliability of the scales was evaluated with Cronbach's alpha coefficient. The level of statistical significance was accepted as p<0.05 (Table 1).

**Table 1:** Distribution of the premenstrual syndrome scores of the students.

PMSS sub-dimensions	X ± SD	Minimum	Maximum
Depressive mood	20.91±5.39	7	35
Anxiety	16.56±4.95	6	30
Fatigue	17.81±4.61	6	30
Irritability	14.53±4.25	5	25
Depressive thoughts	20.09±5.31	7	35
Pain	8.78±2.77	3	15
Appetite changes	9.07±2.92	3	15
Sleep changes	8.94±2.66	3	15
Bloating	8.67±2.96	3	15
Total PMSS	125.40±25.41	56	215
PMSS		N	%
Present		446	72.4
Absent		170	27.6

PMSS: Premenstrual Syndrome Scale.

## RESULTS

The PMS scale total mean score was found to be 125.40±25.41, and the incidence of PMS was determined to be 72.4%.

The age of the students, father's education level, and menstruation duration were determined to be variables affecting PMS, and the difference between the groups was statistically significant (p<0.05). Students in the age group of 17-20 years, whose fathers were high-school graduates, and students whose menstruation lasted 9 days or more were found to have PMS. The average score of those students was higher than that of other groups (Table 2).

**Table 2:** Comparison of personality characteristics of students according to premenstrual syndrome status.

CPS	PMSS		Test/p
	Present	Absent	
Extroversion/introversion	25.55±4.79	26.31±5.15	0.077
Emotional balance/neuroticism	20.88±4.54	17.42±5.43	**0.019**
Consistent/inconsistent	17.02±4.93	12.65±5.35	**0.042**

Bold indicates *p*<0.05.

Of those experiencing PMSS, the mean score was determined as 25.55±4.79 in the extroversion/introversion dimension, 20.88±4.54 in the emotional balance/neuroticism dimension, and 17.02±4.93 in the consistent/inconsistent dimension. A statistically significant relationship was found between the emotional balance/neuroticism dimension and consistent/inconsistent personality and PMS scores of the students participating in the study (Table 3).

**Table 3:** Predictive factors of female students’ perceptions of premenstrual syndrome.

Variables	B (95%CI)	SE	β	T	p
Constant	100.797	8.27		12.176	**0.000**
Age	0.385	1.806	0.008	0.213	0.831
Father's education level	-0.590	1.268	-0.016	-0.466	0.642
Menstrual frequency	-0.951	1.433	-0.023	-0.663	0.507
Extroversion/introversion	-0.824	0.184	-0.159	-4.485	**0.000**
Emotional balance/neuroticism	1.815	0.200	0.361	9.069	**0.000**
Consistent/inconsistent	0.784	0.185	0.167	4.233	**0.000**

R=0.504, Adj. R^2^=0.247, F=34.560, p<0.001. Adj. R^2^: adjusted R square; B: partial regression coefficient; β: standard partial regression coefficient; 95%CI: 95% confidence interval. Bold indicates *p*<0.05.

The predictive power of the linear regression model calculated using the backward elimination method (adjusted R^2^) was determined to be 24.7%. Extrovert/introvert personality trait (β=-0.159), Emotional balance/Neuroticism (β=0.361), and Consistent/inconsistent personality trait (β=0.167) were found to be significant predictors of students experiencing PMS (p<0.05).

## DISCUSSION

Premenstrual syndrome affects the lives of women of reproductive age, with prevalence varying from country to country. The results of this study showed that the average PMSS score was 125.40±25.41 and the frequency of PMS was 72.4%. In a systematic review and meta-analysis study conducted to determine the frequency of menstrual cycle disorders in university students in all geographical regions of the world, the prevalence of PMS was determined as 51.3%^13^. In other systematic review and meta-analysis studies, the prevalence of PMS has been reported to be 70.8% in Iran^14^, 53% in Ethiopia^8^, 43% in India^3^, and 52.2% in Turkey^7^. The prevalence of PMS among university students in Turkey has been reported to vary between 33 and 91.8%^5,7,15–19^. It is thought that these differences in the prevalence of PMS may be due to unequal genetic, dietary, and lifestyle factors among young adult women and various socially accepted practices during the premenstrual and menstrual period^20,21^.

The results of the current research showed that students exhibited the personality trait of being extroverted/introverted more than that has been reported in other studies^22,23^. The extrovert personality traits include being open to communication, energetic, warm-blooded, sociable, talkative, enthusiastic, and excited. It is known that these people are successful and happy in establishing ties with their environment and in business and family life. Individuals with introvert personality traits are calm, introverted, and non-social individuals^24^. Some personality traits may cause intense reactions to PMS-related complaints^10^. In this study, it was determined that the extrovert/introvert personality trait affected PMS, but there appears to be no consensus on this issue in the literature. Some studies have found that being extroverted/introverted did not affect PMS^22,23^, while others have reported that PMS is less common in those with high levels of extroversion^25,26^.

In this study, the emotional balance/neuroticism rates were found to be similar to the rates in other studies^22,23^. Neuroticism is directly related to depression and can cause both adaptation and health problems. Individuals with emotionally balanced personality traits are defined as people who are relaxed, have a high level of self-confidence, and are patient. Neurotic individuals are described as anxious, angry, introverted, and insecure. The results of this study regarding the predictive effect of personality traits on PMS showed that the emotional balance/neuroticism sub-dimension predicts PMS at a significant level. A previous study conducted in Spain showed that the frequency of PMS was higher in people with neurotic personality traits^27^. A relationship between PMS and neurotic personality traits has also been reported in Nigerian university students, and PMS was seen to be significantly more common in women exhibiting neurotic personality traits^28^. From the personality traits sub-dimension, the trait of being consistent/inconsistent refers to a situation being persistent and not contradictory. In this study, consistent personality trait was determined as a predictive variable of PMS. Ölçer et al. evaluated university students and found a significant difference between the PMS and CPS subscale consistent/inconsistent, similar to the current research.

### Strengths and limitations

It is thought that the results obtained from this study will guide healthcare professionals in protecting the mental health of university students experiencing PMS. However, this study also had some limitations. As the study was conducted in a single university, it cannot be generalized to all students experiencing PMS. Another limitation of the study was that because it was a cross-sectional study, a causal relationship could not be established. Therefore, there is a need for further studies with large samples to examine the causal relationship and variables between PMS and personality traits in university students.

## CONCLUSION

The results of this study demonstrated that two-thirds of the students experienced PMS and that personality traits and PMS are interrelated dynamics.
